# Normative data for the Pyramids and Palm Trees Test in literate Persian adults

**Published:** 2018-01-05

**Authors:** Azar Mehri, Seyede Zohreh Mousavi, Mohammad Kamali, Saman Maroufizadeh

**Affiliations:** 1 Department of Speech Therapy, School of Rehabilitation, Tehran University of Medical Sciences, Tehran, Iran; 2 Rehabilitation Research Center, Tehran University of Medical Sciences, Tehran, Iran; 3 Department of Epidemiology and Reproductive Health, Reproductive Epidemiology Research Center, Royan Institute for Reproductive Biomedicine, Academic Center for Education, Culture and Research, Tehran, Iran

**Keywords:** Semantics, Memory and Learning Tests, Reference Values, Iran, Adult

## Abstract

**Background:** Semantic test of Pyramids and Palm Trees (PPT) is the most common test for assessing memory. Since this test is related to language and culture, normative data in different populations are needed.

**Methods:** This study was conducted on 270 literate men and women Persian adults aged from 20 to 69 years. Subjects must select a picture or word between two pictures or words that was closer to target.

**Results:** The word score was significantly positively correlated with the picture score (r = 0.508, P < 0.001). Word scores (median = 50, Q_1_-Q_3_ = 49-51) were higher than the picture scores (median = 50, Q_1_-Q_3_ = 48-51), although the difference was small (P < 0.001).

**Conclusion:** Demographic variables such as age, gender, and level of education were not significant predictors for both versions in Persian population.

## Introduction

Semantic memory indicates common actual knowledge between society members similar to information in a dictionary.^1^ Researchers have used different means to evaluate the semantic system and semantic relations including Camel and Cactus test, the picture-word matching, picture naming, category fluency, phonemic fluency, synonym judgment for words, double description, word checking, class name selection, picture group naming, concrete and abstract word synonym test, environmental sounds test, and target class and association test.^2^^-^^7^ Considering semantics, in patients with aphasia and other neurological disorders, to investigate exact semantic processing it is essential to design different semantic tests. Several studies have shown that the Pyramids and Palm Trees (PPT) test is suitable for this purpose.^3^^,^^8^^-^^10^ Normative data of this test was conducted in different populations such as English,^11^ Spanish,^12^ Italian,^13^ Quebec-French,^14^ and Chinese.^15^

PPT test was designed in 1992 by Howard and Patterson. Since one item of this test is PPT, it is known as PPT test. PPT is a semantic memory test that is used to assess cognition in brain disorders, semantic dementia, Alzheimer’s disease and aphasia. This test measures accessibility to semantic features of words and picture information. It contains 52 sets of words or pictures and was designed for the age range of 18-80 years old. There was no time limit to perform the test and it was conducted individually. The maximum score was 52.^11^

Several studies used PPT to investigate semantic memory in various populations which were mentioned for normative studies. Gudayol-Ferre, et al. investigated the effects of demographic factors (age, gender, and level of education) on PPT test in a Spanish population. The results showed formal education was effective; but there was no meaningful difference in various ages and for both genders.^12^ In Gamboz, et al. study, age and education level were important factors in PPT score.^13^ Klein and Buchanan suggested that PPT is a nonverbal measure of semantic memory that has been frequently used in previous aphasia, agnosia, and dementia research. They concluded that the validity of this test can only be used for clinical purposes and the test-retest reliability was poor.^16^ Normative data for PPT in the French population was obtained by Callahan, et al. Significant association with performance was observed in both level of education and age, but gender had no significant effect.^14^ Guo, et al. studied PPT and other semantic tests in a Chinese population. Their results showed that the level of education and age affected the performance.^15^ PPT test was standardized in England by Howard and Patterson on 73 healthy adults and all participants performed well in this test. They did not have more than 3 errors and their scores were above 90%. The score of less than 47/52 in this test was a sign of semantic memory impairment. They found that level of education has stronger effect on test scores rather than age and gender.^11^

The study of semantic abilities and disabilities on patients with brain lesion can provide important information about semantic representation and processing. It is possible that semantic representation is intact in a patient who cannot name an object, but he/she has difficulty in phonological access. PPT test can evaluate semantic ability without involving the phonological access. Considering the fact that the test depends on language and culture, normative data in different populations is necessary. This study aimed to standardize PPT test data in Persian speakers. Therefore, this study was conducted on 20- to 69-year-old healthy Iranians of both genders.

## Materials and Methods

The participants included 270 healthy Persian adults (126 men and 144 women) within the age range of 20 to 69 years. The age range was divided into five groups: 20-29, 30-39, 40-49, 50-59, and 60-69 years. Level of education was also divided into three levels: 1-11 years, 12-14 years, and 15 years or more. Mini-Mental State Examination (MMSE) score in 99.6% of subjects was over 27, and was 25 in the remaining 0.4%; in which all of them were within the normal range of MMSE.^17^

**Figure 1:**
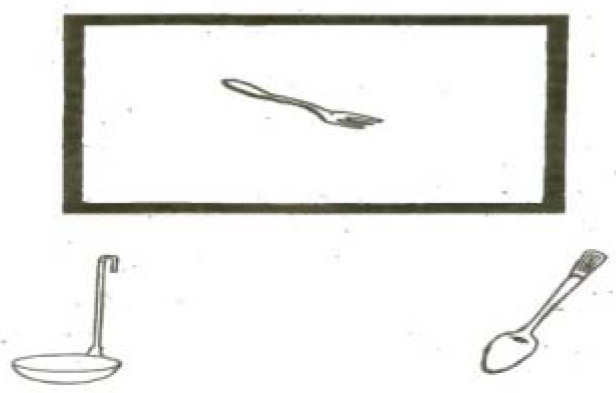
Picture sample of Pyramids and Palm Trees (PPT) test

PPT consists of 52 sets, each one containing 3 words or pictures, and also 3 samples in order to introduce the aim of the test to subjects. The target word or picture was placed on the top of the other 2 words or pictures. The subject was asked to match the target word or picture with one of the words or pictures below. Each of the 3 words or pictures on a sheet was offered to subjects; and he/she was asked to choose one word or picture which was closer to the target item, and point it (Figure 1). The maximum score of the test is 52.

After accessing to the English version of PPT, it was translated into Persian. In order to evaluate face validity of the test, the picture version was given to 6 speech and language pathologists, and a pilot study was done on 11 adults older than 50 years of age with different levels of education. Finally, cultural adaptation was performed according to experts’ suggestions and the results of the pilot study. Subsequently, some changes were made on the pictures that were rather apart from the Persian culture such as: canoe to boat, tulip to wheat, nun to clergyman, church to mosque, bellows to hand fan, and ticket to Persian ticket. Additionally, due to cultural differences, one set including Acorns-donkey-pig was moved to the example of the test and instead another set including waistcoat-bowtie-necklace was added to other sets of the test. Some minor changes were also made including: a picture of the thimble was drawn smaller; kayak was drawn bigger; pictures of saddle, slippers and puddle were slightly changed; and in the picture of the pyramid, another small pyramid was added. Afterwards, the Persian word version was changed based on the new picture version.

The new version of Persian PPT (P-PPT) was presented to 14 experts (11 speech and language pathologists and 3 linguists), and they were asked to rate the familiarity and clarity of the changed pictures on a scale of 1 to 5, that 1 showed lowest and 5 the highest familiarity and clarity. Then, they were asked to assess the ability of semantic relations of three pictures in each page on a scale of 1 to 5. In this study, content validity rate (CVR) was calculated using Lawshe formula and appropriate pictures were chosen according to 11 experts’ suggestions. Finally, the score 57% was accepted for the final version of test.^18^

Lawshe formula: CVR = (Ne – N/2)/(N/2); Ne: Numbers of experts have given a score of 5 to each question, N: total number of experts. 

To perform P-PPT, at first, questionnaires including demographic information and informed consents were completed by participants. MMSE scores were obtained for each subject. The best cut-off point of MMSE was 23.^17^

The Inclusion criteria for participants were: a) being 20 to 69 years old, b) being Persian native speaker, c) being literate, d) having no history of neurological or psychiatric disorders, e) having no cognitive deficits (based on MMSE), f) having no audio-visual problems, and g) having no history of drug abuse. Lack of cooperation and failure to complete the word version was a criterion for exclusion. It should be mentioned that, at the first step, picture version of the test was performed, and then after 7 to 10 days the word version was assessed. The test was performed according to the instruction of PPT. No time limit was set for performing the test. Correct and incorrect responses were recorded by the examiner and the total score was calculated based on the number of correct answers. Each correct response was given 1 point.

Statistical analyses were conducted using SPSS software (version 21, IBM Corporation, Armonk, NY, USA). Descriptive statistics for continuous variables were presented as mean ± standard deviation (SD) or median (interquartile range), and for categorical variables as numbers (percentages). Nonparametric tests were chosen, as P-PPT scores were not normally distributed (Shapiro-Wilk test). The relationships between demographic characteristics (sex, age group, level of education) and PPT were explored using Mann-Whitney and Kruskal-Wallis tests. Spearman correlation coefficient was used to examine the relationship between word and picture scores of P-PPT; moreover, the word and picture scores of P-PPT were compared using Wilcoxon signed-rank test. All statistical tests were two-tailed, and the level of statistical significance was set at less than 0.05 (P < 0.050).

## Results

According to Lawshe formula, content validity rate was over 57%. Demographic characteristics of 270 participants are shown in table 1.

**Table 1:** Demographic characteristics of the participants (n = 270)

**Variable**	**n (%)**
Sex	
Men	126 (46.7)
Women	144 (53.3)
Age group	
20-29 years	57 (21.1)
30-39 years	53 (19.6)
40-49 years	58 (21.5)
50-59 years	52 (19.3)
60-69 years	50 (18.5)
Level of education	
Primary (1-11 years)	57 (21.1)
Secondary (12-14 years)	96 (35.6)
University (≥ 15 years)	117 (43.3)

As shown in table 2, the mean ± SD score of word version of PPT was 50.07 ± 1.80, and the median (range) was 50 (49-51). The mean ± SD score of picture version of PPT was 49.26 ± 2.67, and the median (range) was 50 (48-51). The relationships between the demographic characteristics (sex, age, level of education) and P-PPT scores using univariate analysis are shown in table 2. According to Mann-Whitney test, scores of the word version of P-PPT were not correlated with sex (P = 0.059). Word scores also did not differ significantly by the age groups (P = 0.350), and level of education (P = 0.921). Moreover, same results were obtained for picture version of PPT.

**Figure 2:**
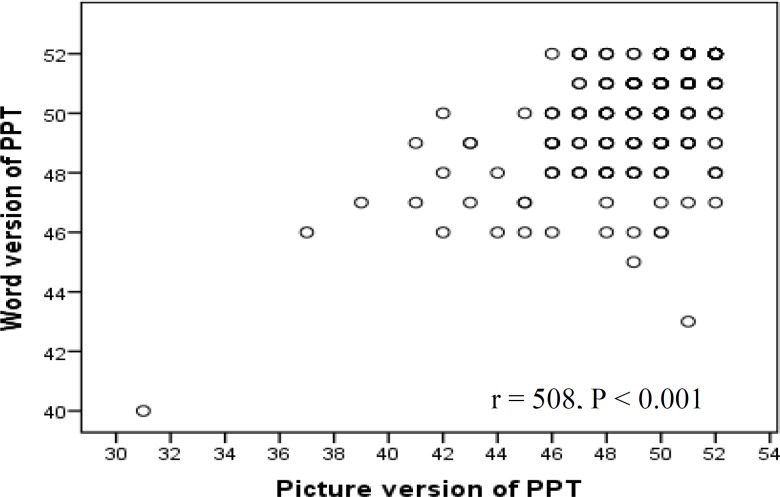
Scatter plot of word score versus picture score of Persian Pyramids and Palm Trees (P-PPT) test

Spearman correlation coefficient was used to examine the relationship between word and picture scores. As expected, the word score was significantly positively correlated with the picture score (r = 0.508, P < 0.001) (Figure 2).

**Table 2:** Relationship between Persian Pyramids and Palm Trees (P-PPT) scores and demographic characteristics of participants

**Variable**	**Picture**			**Word**		
	**Mean ± SD**	**Median (Q**_1_ **-Q**_3_ **)**	**P**	**Mean ± SD**	**Median (Q**_1_ **-Q**_3_ **)**	**P**
Sex			0.693*			0.059*
Men	49.17 ± 2.84	50 (48-51)		50.26 ± 1.82	51 (49-52)	
Women	49.34 ± 2.52	50 (48-51)		49.91 ± 1.77	50 (49-51)	
Age group			0.214**			0.350**
20-29 years	49.81 ± 1.85	50 (48.5-51)		50.32 ± 1.70	50 (49-52)	
30-39 years	49.25 ± 2.35	50 (48-51)		49.81 ± 1.73	50 (48.5-51)	
40-49 years	49.64 ± 2.02	50 (48.75-51)		50.33 ± 1.53	51 (50-51)	
50-59 years	48.92 ± 2.11	49 (47.25-50)		49.90 ± 1.74	50 (49-51)	
60-69 years	48.58 ± 4.35	50 (48-51)		49.96 ± 2.27	51 (49-52)	
Education level			0.171**			0.921**
Primary (1-11 years)	48.91 ± 3.10	50 (48-51)		50.14 ± 1.56	50 (49-51)	
Secondary (12-14 years)	49.11 ± 2.75	50 (48-51)		49.92 ± 2.13	50.5 (49-51)	
University (≥ 15 years)	49.56 ± 2.36	50 (48.5-51)		50.17 ± 1.61	50 (49-52)	

SD: Standard deviation, Q_1_ = First quartile, Q_3_ = Third quartile

* Mann-Whitney test,

** Kruskal-Wallis test

**Figure 3:**
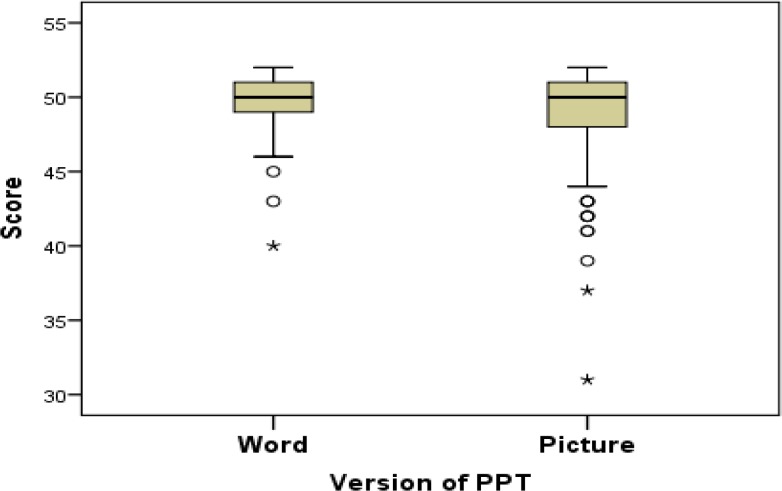
Comparison between word and picture scores of Persian Pyramids and Palm Trees (P-PPT) test

The word and picture scores of PPT were compared using Wilcoxon signed-rank test. As it is shown in figure 3, word scores (median = 50, Q_1_-Q_3_ = 49-51) were higher than the picture scores (median = 50, Q_1_-Q_3_ = 48-51); although the difference was small (P < 0.001).

## Discussion

Nature of meaning closely depends on categorization as a concept determines how the things are related. Concepts are mental representations of categories and they can help based on common characteristics. 

There are 2 types of semantic tasks:

1. Category sorting (that is related to semantic categories e.g. summer versus winter clothing);

2. Matching pictures based on semantic relationships (e.g. rabbit: carrot).^19^

Recent study of semantic memory organization on patients with brain lesion has shown that there are double dissociations between different types of tasks (picture and words), different types of words (concrete and abstracts), and different types of semantic features (visual and functional).^1^

In 1988, Shallice suggested that visual and verbal systems and their processing are distinct.^1^ In 1992, Caramazza, Hillis, Rapp, and Romani summarized four hypotheses about semantic systems for visual and verbal materials based on previous studies.^1^

One of these hypotheses is about content organization within a single semantic system. This hypothesis states that semantic predicates that have high correlation also have stronger links than others. Accessing semantic information links of objects is possible through words and pictures. Authors argue that pictures are directly associated with perceptual features while words are indirectly associated with component of semantic information.^1^

Generally, there is no strong evidence to show a difference between semantic systems of picture and word representations.

In picture version, as there was no significant difference in among different age groups (in both genders), it is concluded that increasing age has no effect on the semantic relation in pictures. These findings are consistent with Gudayol-Ferre, et al. study, as they also find no significant difference between the total PPT score and age;^12^ but the findings do not support the results of Gamboz, et al.,^13^ Callahan, et al.,^14^ and Guo, et al.;^15^ because their results showed a significant impact on picture version between age groups. Although the age groups of Callahan, et al. study (i.e. 19-39, 40-49, 50-59, 60-69, and over 70 years),^14^ and Guo, et al.’s study (i.e. 20-80)^15^ were similar to our study, the results were different. In Gamboz, et al. study,^13^ 464 healthy adults (226 men and 238 women) from the age of 49 to 94 years participated. The number of subjects and their age groups can be of the one causes of different results to our study because their participants were older. Their results indicated the significant influence of age on picture version (P < 0.010) and word version (P < 0.010).

There was no significant difference between genders for the mean score of the picture version. This finding is similar to Gudayol-Ferre, et al.,^12^ Gamboz, et al.,^13^ Callahan, et al.,^14^ and Guo, et al.^15^ It can be concluded that the gender factor is not effective in this semantic test. 

There was no significant difference in the mean score of the picture version for level of education. In other words, the score of people who had higher than 15 years of education had no significant difference to people with other two levels of education. This finding is not consistent with Gudayol-Ferre, et al.,^12^ Gamboz, et al.,^13^ Callahan, et al.,^14^ Howard,^11^ and Guo, et al.^15^ studies; almost in all the highlighted studies, the level of education was divided into two main groups: a) below the level of university education (i.e. > 12 years) and, b) higher than (i.e. < 12 years). However, in Gudayol-Ferre, et al. study, they considered three levels of education: 1-5, 6-11, and 12 years and more, and suggested that there was a significant difference between low education and their score while they found no significant difference between two other levels of education and obtained scores.^12^

In general, it has been seen that people with lower education had poor performance but in our study, this result does not find. This may be due to different sample sizes in various levels of education (less than 12 years: 21.1%, 12-14 years: 35.6%, and 15 years and more: 43.3%) and the different educational system in Iran. It seems that picture retrieval was easy for low education level in P-PPT.

In word version, significant difference was not discovered between the mean score and age groups. It seems that subject is familiar with semantic relations. While in most studies, the picture version was investigated, Gamboz, et al.^13^ stated that there is no significant difference in word version, that is similar to the results of the current study.

The average scores investigated between men and women did not show a significant difference. 

Also these findings showed no significant differences between age, gender, and level of education in the Persian word version similar to picture version. 

In both versions, our findings suggest that the mean scores of picture and word versions are different, and picture score is lower than word score. It may be that picture items of the test are very close together and people experience the error visually. Besides, familiarity to picture version can have an effect on word version responses.

Finally, semantic hypothesis review indicated that there was association between pictorial and verbal representations. Furthermore, this study demonstrated the same correlation, as the picture score was high, and the word score was even better. While, word preference was higher than picture preference in our results; which is against the content organization within single semantic system hypothesis.

## Conclusion

Previous studies showed that the performance of both picture and word versions is affected by demographic variables such as age, gender, and level of education, and these factors were significant predictors for both versions; but in Persian population, they were not significant. Therefore, the subject scores should be interpreted according to all affective factors.

## Notes:

**How to cite this article:** Mehri A, Mousavi SZ, Kamali M, Maroufizadeh S. Normative data for the Pyramids and Palm Trees Test in literate Persian adults. Iran J Neurol 2018; 17(1): 18-23.
